# Supplementary material for: Machine Learning Accelerates Crystallization for Structure Determination
Source: Angew Chem Int Ed Engl. 2026 May 4;65(25):e1218503. doi: 10.1002/anie.1218503 (PMC13266958; doi:10.1002/anie.1218503)

checkCIF/PLATON report

Structure factors have been supplied for datablock(s) lcz021

THIS REPORT IS FOR GUIDANCE ONLY. IF USED AS PART OF A REVIEW PROCEDURE FOR PUBLICATION, IT SHOULD NOT REPLACE THE EXPERTISE OF AN EXPERIENCED CRYSTALLOGRAPHIC REFEREE.

No syntax errors found.   CIF dictionary   Interpreting this report

Datablock: lcz021

Bond precision:	C-C = 0.0070 A		Wavelength=1.54184
Cell:	a=8.4178(2)	b=12.5046(2)	c=16.8597(3)
	alpha=102.357(2)	beta=101.656(2)	gamma=102.699(2)
Temperature:	100 K		

	Calculated	Reported
Volume	1632.36(6)	1632.36(6)
Space group	P -1	P -1
Hall group	-P 1	-P 1
Moiety formula	C25 H15 Ag3 F18 N6 O3	C15 H3 Ag3 F18 N6, C10 H12 O3
Sum formula	C25 H15 Ag3 F18 N6 O3	C25 H15 Ag3 F18 N6 O3
Mr	1113.04	1113.04
Dx,g cm-3	2.264	2.265
Z	2	2
Mu (mm-1)	15.655	15.655
F000	1068.0	1068.0
F000’	1073.21	
h,k,lmax	10,15,21	10,15,21
Nref	7006	6608
Tmin,Tmax	0.058,0.209	0.117,1.000
Tmin’	0.004	

Correction method= # Reported T Limits: Tmin=0.117 Tmax=1.000
AbsCorr = MULTI-SCAN

Data completeness= 0.943 Theta(max)= 78.334

R(reflections)= 0.0410(6355) wR2(reflections)= 0.1159(6608)

S = 1.068 Npar= 499

The following ALERTS were generated. Each ALERT has the format
test-name_ALERT_alert-type_alert-level.
Click on the hyperlinks for more details of the test.

Alert level C

```
PLAT042_ALERT_1_C Calc. and Reported MoietyFormula Strings  Differ     Please Check
              Calc: C25 H15 Ag3 F18 N6 O3
              Rep.: C15 H3 Ag3 F18 N6, C10 H12 O3
PLAT213_ALERT_2_C Atom F00P            has ADP max/min Ratio .....        3.2 prolat
PLAT213_ALERT_2_C Atom F00S            has ADP max/min Ratio .....        3.4 prolat
PLAT213_ALERT_2_C Atom F011            has ADP max/min Ratio .....        3.4 prolat
PLAT213_ALERT_2_C Atom F015            has ADP max/min Ratio .....        4.0 prolat
PLAT213_ALERT_2_C Atom F01J            has ADP max/min Ratio .....        3.9 prolat
PLAT220_ALERT_2_C NonSolvent   Resd 1  F   Ueq(max)/Ueq(min) Range        4.4 Ratio
PLAT911_ALERT_3_C Missing FCF Refl Between Thmin & STh/L=    0.600         50 Report
               -1  1  0,   1  3  0,  -9  5  0,  -2 12  0,  -4-11  1,   0 -1  1,
               -1  0  1,   0  1  1,  -7  5  1,   2-13  2,  -2 -2  2,  -1  1  2,
               -2  2  2, -10  3  2, -10  4  2, -10  5  2,  -1  7  2,   4 10  2,
               -9 -2  3,   2  0  3, -10  2  3, -10  3  3,  -9  3  3, -10  4  3,
               -9  4  3, -10  5  3,   0 -3  4,   5  0  4, -10  2  4, -10  3  4,
                   (   20 More Missing: see the .ckf listing file)
PLAT977_ALERT_2_C Check Negative Difference Density on H016      .      -0.36 eA-3
```

Alert level G

```
PLAT003_ALERT_2_G Number of Uiso or U(i,j)  Restrained non-H-Atoms          4 Report
PLAT083_ALERT_2_G SHELXL Second Parameter in WGHT  Unusually Large       5.05 Why ?
PLAT154_ALERT_1_G The s.u.'s on the Cell Angles are Equal ..(Note)      0.002 Degree
PLAT186_ALERT_4_G The CIF-Embedded .res File Contains ISOR Records          3 Report
PLAT242_ALERT_2_G Low    'MainMol' Ueq as Compared to Neighbors of       C00V Check
PLAT242_ALERT_2_G Low    'MainMol' Ueq as Compared to Neighbors of       C014 Check
PLAT242_ALERT_2_G Low    'MainMol' Ueq as Compared to Neighbors of       C01D Check
PLAT242_ALERT_2_G Low    'MainMol' Ueq as Compared to Neighbors of       C01F Check
PLAT434_ALERT_2_G Short Inter HL..HL Contact F00O     ..F00O     .       2.76 Ang.
                                              1-x,1-y,2-z  =      2_667 Check
PLAT720_ALERT_4_G Number of Unusual/Non-Standard Labels ..........         70 Note
              Ag01    Ag02    Ag03    F004    F005    F006    F007    F008
              F009    F00A    F00B    F00C    O00D    N00E    N00F    F00G
              N00H    O00I    N00J    F00K    N00L    N00M    O00N    F00O
              F00P    F00Q    C00R    H00R    F00S    C00T    C00U    C00V
              C00W    C00X    H00X    C00Y    C00Z    C010    H010    F011
              C012    H012    C013    C014    F015    C016    H016    C017
              C018    H01A    H01B    H01C    C019    C01A    H01D    H01E
              H01F    C01B    C01C    C01D    C01E    C01F    C01G    C01H
              H01G    H01H    H01I    C01I    H01J    F01J
PLAT860_ALERT_3_G Number of Least-Squares Restraints ............          24 Note
PLAT883_ALERT_1_G Absent Datum for _atom_sites_solution_primary ..     Please Do !
```

```
PLAT912_ALERT_4_G Missing # of FCF Reflections Above STh/L=  0.600        347 Note
PLAT913_ALERT_3_G Missing # of Very Strong Reflections in FCF ....          2 Note
                1  3  0,  -2  2  2,
PLAT941_ALERT_3_G Average HKL Measurement Multiplicity ..........        2.0 Low
PLAT969_ALERT_5_G The 'Henn et al.' R-Factor-gap value ..........      4.580 Note
              Predicted wR2: Based on SigI**2  2.53 or SHELX Weight 10.86
PLAT978_ALERT_2_G Number C-C Bonds with Positive Residual Density.          0 Info
```

```
   0 ALERT level A = Most likely a serious problem - resolve or explain
   0 ALERT level B = A potentially serious problem, consider carefully
   9 ALERT level C = Check. Ensure it is not caused by an omission or oversight
  17 ALERT level G = General information/check it is not something unexpected

   3 ALERT type 1 CIF construction/syntax error, inconsistent or missing data
  15 ALERT type 2 Indicator that the structure model may be wrong or deficient
   4 ALERT type 3 Indicator that the structure quality may be low
   3 ALERT type 4 Improvement, methodology, query or suggestion
   1 ALERT type 5 Informative message, check
```

Validation response form

Please find below a validation response form (VRF) that can be filled in and pasted into your CIF.

```
# start Validation Reply Form
_vrf_PLAT042_lcz021
;
PROBLEM: Calc. and Reported MoietyFormula Strings  Differ     Please Check
RESPONSE: ...
;
_vrf_PLAT213_lcz021
;
PROBLEM: Atom F00P            has ADP max/min Ratio .....        3.2 prolat
RESPONSE: ...
;
_vrf_PLAT220_lcz021
;
PROBLEM: NonSolvent   Resd 1  F   Ueq(max)/Ueq(min) Range        4.4 Ratio
RESPONSE: ...
;
_vrf_PLAT911_lcz021
;
PROBLEM: Missing FCF Refl Between Thmin & STh/L=    0.600         50 Report
RESPONSE: ...
;
_vrf_PLAT977_lcz021
;
PROBLEM: Check Negative Difference Density on H016      .      -0.36 eA-3
RESPONSE: ...
;
# end Validation Reply Form
```

It is advisable to attempt to resolve as many as possible of the alerts in all categories. Often the minor alerts point to easily fixed oversights, errors and omissions in your CIF or refinement strategy, so attention to these fine details can be worthwhile. It is up to the individual to critically assess their own results and, if necessary, seek expert advice.

PLATON version of 17/09/2025; check.def file version of 17/09/2025

---

duplicate check

No duplication found

Datablock lcz021 - ellipsoid plot

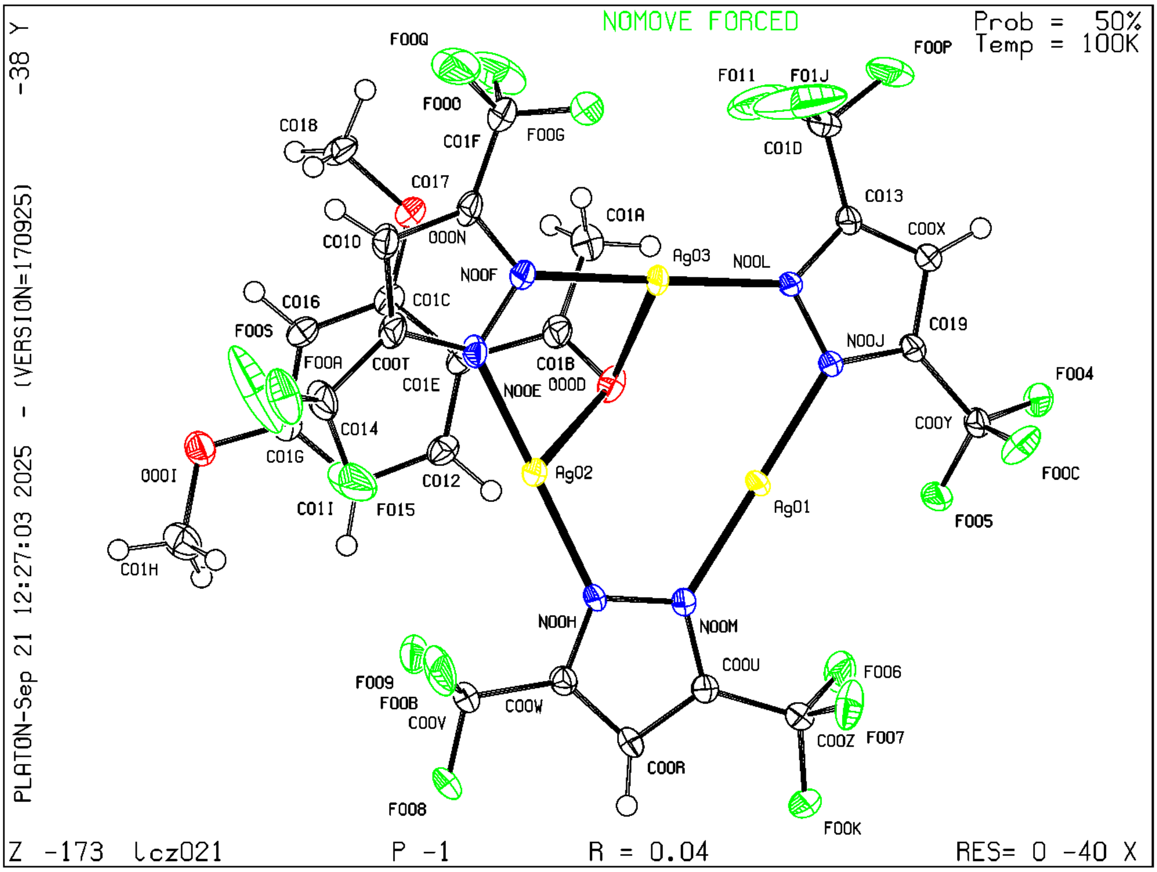

Supplement: Supplementary file 2 — Supporting File 2: anie72462‐sup‐0002‐DataFile.zip. [file ANIE-65-e1218503-s002.zip › supplementary_materials_crystallographic_data submit.zip!/cif_and_checkcif(1-114)/Ag3Pz3_21/Ag3Pz3_21_checkcif.pdf]
